# Constipation: A Pristine Universal Pediatric Health Delinquent

**DOI:** 10.7759/cureus.52551

**Published:** 2024-01-19

**Authors:** Kona Chowdhury, Susmita Sinha, Santosh Kumar, Mainul Haque, Rahnuma Ahmad

**Affiliations:** 1 Department of Pediatrics, Gonoshasthaya Samaj Vittik Medical College, Dhaka, BGD; 2 Department of Physiology, Khulna City Medical College and Hospital, Khulna, BGD; 3 Department of Periodontology and Implantology, Karnavati School of Dentistry, Karnavati University, Gandhinagar, IND; 4 Karnavati Scientific Research Center (KSRC), Karnavati School of Dentistry, Karnavati University, Gandhinagar, IND; 5 Department of Pharmacology and Therapeutics, National Defence University of Malaysia, Kuala Lumpur, MYS; 6 Department of Physiology, Medical College for Women and Hospital, Dhaka, BGD

**Keywords:** fast food, fruits, low intake, consumption, issue, public health, global, dietary fiber, constipation, childhood

## Abstract

Constipation suffered by children is a global public health problem. Functional constipation (FC) brings about deteriorating effects in the children's lives who suffer from it. The risk factors for the development of constipation include the consumption of a diet low in fiber and high in calories (such as the consumption of fast food), a sedentary lifestyle with a lack of exercise, a family history of constipation, and emotional and psychological stress endured by children in their families. It is one of the most common causes of stomachaches in children. FC may lead to fecal incontinence (FI), anal fissures, recurrent urinary tract infections (RUTI), and enuresis in children. Severe constipation may result in stool becoming rock-hard and inflexible in the rectum, which is clinically identified as fecal impaction. It is imperative to perform clinical evaluation and treatment, including pharmacological (the use of stimulant and osmotic laxatives) and non-pharmacological (education, changes in diet, intervention to promote positive behavior and address any emotional issues, toilet training, and physiotherapy for the pelvic floor) interventions. In the case of refractory patients, neuromodulation, the irrigation of the anal canal, and surgical management may be needed. It is essential to lead a healthy, stress-free lifestyle with plenty of exercise and a balanced diet rich in fiber (such as fruits and vegetables) so children can have regular bowel habits and thrive.

## Introduction and background

Childhood death due to diarrhea has declined globally [1-4]. The incidence of diarrheal disorders is also declining due to improved sanitation [1,5,6] and rotavirus vaccination [7,8]. Among functional gastrointestinal diseases (FGID) of children, constipation has recently become highly prevalent worldwide [9,10]. This chronic disorder enormously influences the lives of concerned children and family members and healthcare resources [11,12]. Nowadays, childhood constipation (2-4 years of age) [13] is usually diagnosed according to the Rome IV criteria (the term Rome evolved from the Rome Foundation). This foundation works to improve people's lives with disorders of gut-brain interaction. The Rome Foundation is located in Raleigh, North Carolina, USA [9,14]. The Rome IV now distinguishes between children with and deprived of toilet coaching. Children without toilet training may be identified with functional constipation (FC) if they present with a minimum of two of the following criteria: two or fewer evacuations of one's bowels per seven days with a history of unnecessary stool holding, agonizing defecation, and rock-hard fecal matter; large-width stools; and/or the presence of a substantial fecal bulk, which is stuck in the rectum. For those children with toilet training, two added principles are used: as a minimum, one incident of bowel incontinence every seven days and/or a history of colossal-sized feces that often block the toilet [15-17]. The management of constipation is arduous because of its complex pathophysiological nature [14,18]. Most of the time, the cause is unknown and is depicted as functional constipation (FC) [19]. Children suffering from FC is a phenomenal struggle. FC equally affects their custodians or caregivers socially, physically, and emotionally [9]. The management of childhood constipation mainly depends on lifestyle modification and osmotic laxatives [9]. Treatment often fails because of poor compliance with medications because of higher-cost drugs for prolonged intervention and difficulties in altering dietary habits. Therefore, it is challenging to halt the causal nexus [20-24].

Moreover, clinicians also have shown a lack of knowledge of this functional gastrointestinal disorder [25]. Despite being aware of the Rome IV criteria, pediatric caregivers from Arab countries used different definitions for childhood constipation [26]. A study from Thailand found that older pediatricians are more reluctant to advise on non-pharmacological intervention, e.g., fluid intake and toilet training, compared to recently graduated ones [27]. Only 16.4% of pediatricians in Korea had appropriate knowledge of the Rome IV criteria. As a result, significant discrepancies were noticed between the protocol and actual practice [28]. Hasosah et al. (2022) conducted a study in eight Arab countries (Saudi Arabia, Iraq, Lebanon, Oman, Bahrain, the United Arab Emirates {UAE}, Qatar, and Kuwait), revealing improved overall awareness levels regarding the Rome IV criteria. Nonetheless, statistically significant (p<0.001) differences among pediatric clinicians regarding knowledge and practice were observed [26]. Torres et al. (2015) reported that Brazilian pediatricians' knowledge regarding FC therapeutic intervention is not up to the mark [29].

Problem statement

Childhood constipation has become highly prevalent worldwide [9,10], and this condition has a substantial adverse impact on the lives of concerned children and their household members [11,12]. Nonetheless, this functional gastrointestinal disorder is often overlooked.

Objectives of the study

This review highlights the epidemiology of childhood constipation in developed and underdeveloped countries. It aims to create awareness among clinicians about the factors associated with this disorder and the substantial physical, psychological, and economic impact it may exert on children and caregivers.

## Review

Materials and methods

**Figure 1:**
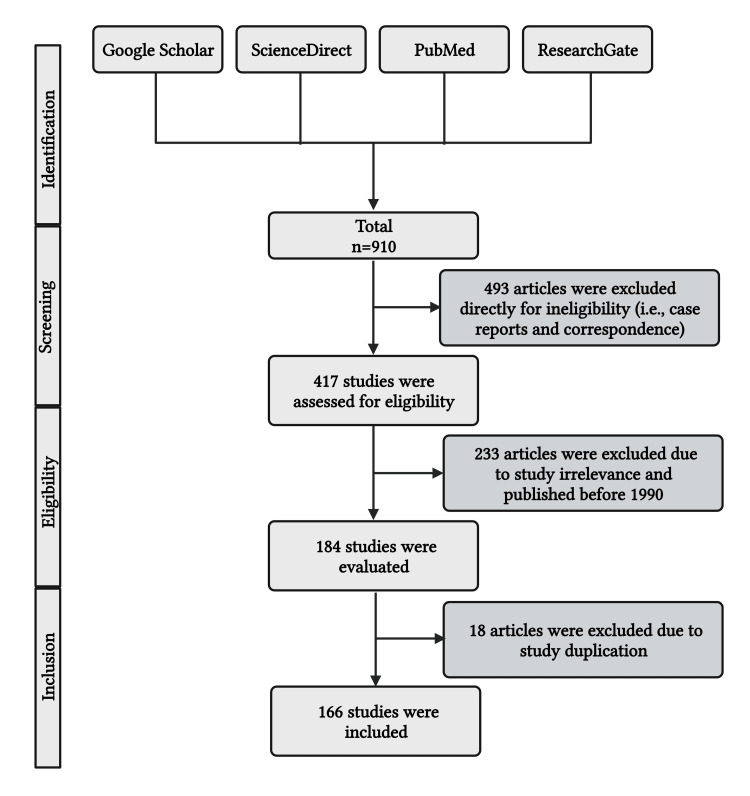
The PRISMA chart illustrates the inclusion of study materials Note: This figure has been drawn with the premium version of BioRender (https://biorender.com, accessed on 12 January 2024) with the license number LV26CTQ2AE. Image credit: Susmita Sinha PRISMA: Preferred Reporting Items for Systematic Reviews and Meta-Analyses

An extensive review of the literature was done to acquire necessary information regarding the prevalence of constipation in children in developed and developing nations. Furthermore, an inclusive literature search was also conducted to determine the level of awareness among doctors on the characteristics linked to this disorder and the significant negative effects it may have on children and caregivers in terms of physical, psychological, and economic aspects. The online archives that were accessed for the scientific literature search were PubMed, ResearchGate, ScienceDirect, and Google Scholar (Figure 1). To find more resources, the reference list of relevant materials was examined. The following keywords were searched to explain this issue: fast food, consumption, fruits, vegetables, dietary fiber, global, constipation, public health, and health implications. We excluded papers published before 2000 that were written in languages other than English. The suitability of the publications was carefully evaluated before their inclusion in the research. Duplicate articles were eliminated. Following the independent evaluation and inclusion of the suggested literary works, a follow-up conversation was arranged to address any controversies, queries, errors, or biases relevant to the work.

Main body of the review paper

Epidemiology

**Figure 2:**
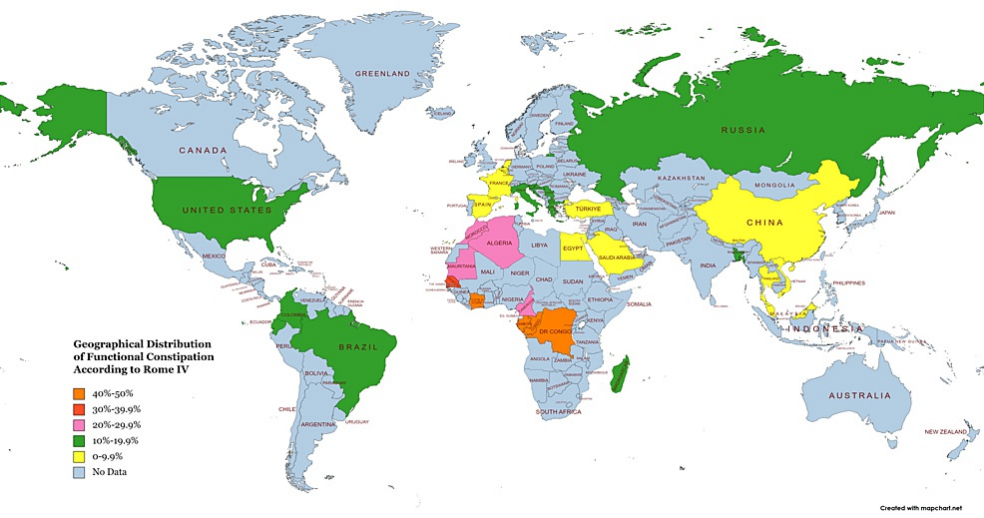
Geographical distribution of functional constipation in children worldwide Note: This figure is created based on Jang et al. [28] with mapchart.net. Image credit: Susmita Sinha

Globally, FC in children is on the rise [19,30,31]. Worldwide, its prevalence ranges from 0.5% to 32.2% (Figure 2) [30,32-36]. In Europe, 16%-19% of children are affected by constipation [37]. The prevalence rate of constipation in Italy is 16.1%; in Germany, it is 6.8%; and in Dutch children, it is 15.6% [38-40]. In Mediterranean countries, the frequency is up to 28.8% [41]. In the USA, 3%-5% of pediatric visits occur due to constipation [42]. It is generally believed that children from the developed world suffer most from constipation, especially from Western countries [43]. The picture has changed with the rise of non-communicable diseases in low- and middle-income countries (LMICs) [44]. According to one study, constipation is prevalent primarily in Africa (31.4%), followed by America (10%-12.1%) [22,45]. In Brazil, the prevalence rate of FC in preschool children is 23% [46], and in infants, it is 7.6% [47]. Columbia (12.5%) and Ecuador (11.8%) shared almost similar prevalence rates [48,49]. Data from Asia is also non-promising. Countries of Asia with developed economies have shown higher frequency. It is 8.4%, 3.9%, 17.1%, 18.1%, and 5.6% in China, Japan, India, Indonesia, and Vietnam, respectively [30,41,50-52]. Developing nations such as Bangladesh and Sri Lanka reported that 19% and 8% of children were affected by this disorder, respectively [53,54]. All these data indicate that constipation in children has become equally prevalent among developed countries and LMICs, making it a global health problem that needs to be addressed soon [43,55,56].

Risk Factors

The lack of fiber is a forefront factor among the well-known risk factors of childhood constipation [57]. The association of fiber in food and constipation has been established by several studies [58-62]. Dietary fibers are a non-digestible form (resistant toward human intestinal enzyme) of composite saccharides, basically cellulose and noncellulosic polysaccharides, for example, hemicellulose, pectic constituents, mucilage, gums, and a noncarbohydrate ingredient lignin. Lignin is a composite polysaccharide polymer that toughens and reinforces the cell wall by binding with cellulose and hemicellulose [63,64]. Lignin is abundant in fruits, such as strawberries and peaches, and whole-grain foods, such as bran, nuts, and seeds [65]. A plant's cell wall is composed of cellulose components [66]. These celluloses containing fibers are widely known as roughage or bulk-forming agents in the human intestine. Additionally, the human intestine cannot digest these fibers; those fibers increase stool volume and weight and soften stool. Therefore, constipated individuals struggle less during bowel movement [67-70].

Dietary fibers are of two types: soluble and insoluble in water [64]. Soluble fibers are again three kinds: non-viscous, readily fermented; viscous/gel-forming, readily fermented; and viscous/gel-forming, nonfermented [71]. Nevertheless, insoluble fiber has only one type: poorly fermented [71]. Soluble fiber comes from diets such as oats, oat bran, barley, apples, citrus fruits, nuts, seeds, beans, lentils, peas, psyllium, carrots, and vegetables [72,73]. The principal sources of insoluble dietary fiber are vegetables, wheat bran, and whole grains [73,74]. There are only two mechanisms that regulate stool formation and improve bowel habits in the large intestine. Those actions are as follows: 1) the bulky/grainy insoluble fiber flecks (e.g., wheat bran) instinctively infuriating the large intestinal gut mucosal layer to secrete water and mucous and 2) the high water-holding competence of gel-growing soluble fiber (e.g., psyllium). After that, it increases stool volume and easy passage [75].

**Figure 3:**
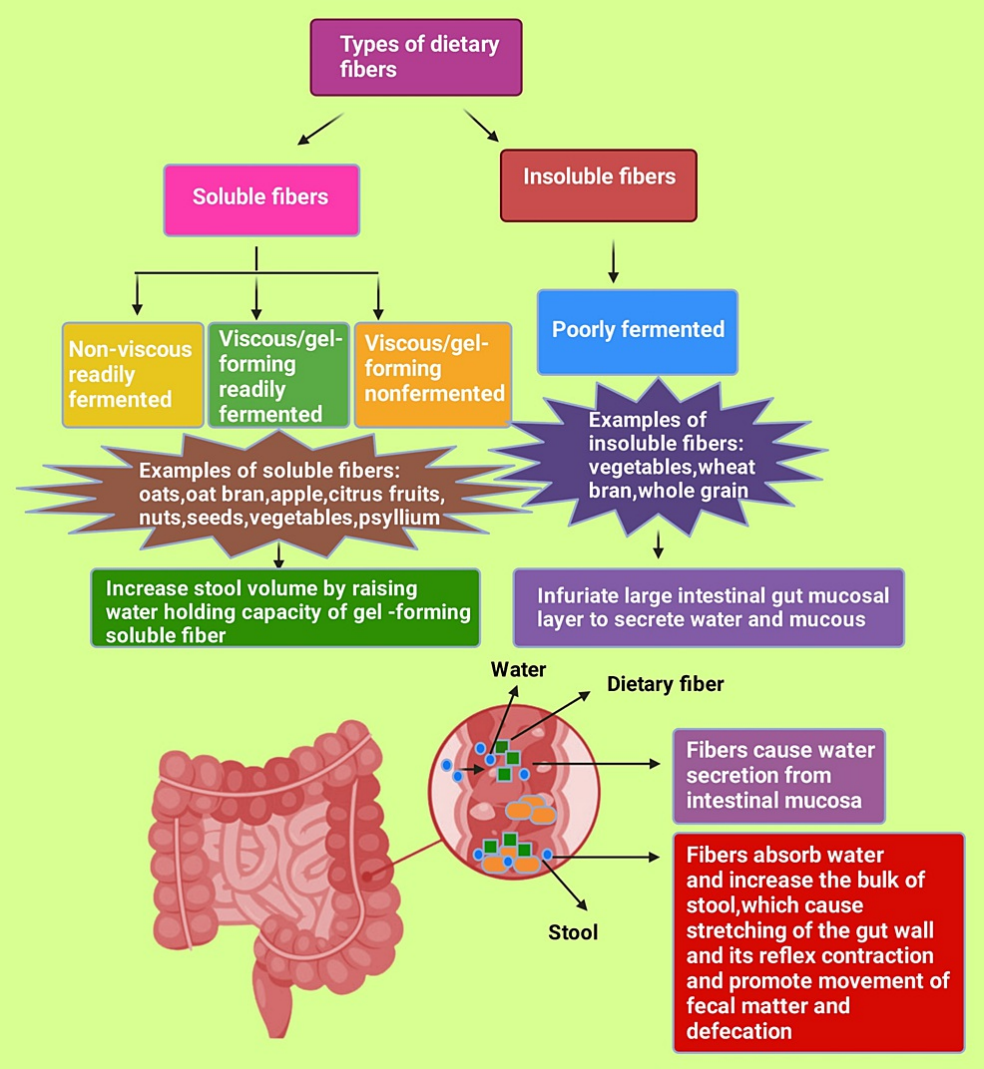
The types of dietary fibers with their examples and the effects of dietary fibers on bowel movement Note: This figure has been drawn with the premium version of BioRender (https://biorender.com, accessed on 28 December 2023) with license number TK26CTFK71. Image credit: Rahnuma Ahmad

The effect of dietary fiber in the small intestine is principally on cholesterol and blood sugar control (Figure 3) [75]. Multiple studies reported that non-digestible carbohydrate-containing dietary fibers reduce the number of non-communicable diseases such as type 2 diabetes mellitus and cardiovascular diseases by minimizing blood cholesterol and glucose [76-81]. Nevertheless, the exact mechanism remains obscure [76,77,79]. One study reported that a higher amount of bile acid synthesis, by contrast to an impediment of cholesterol absorption or origination, is possibly responsible for the cholesterol-reducing pharmacodynamics of barley β-glucan [80]. Another study revealed that soluble dietary fiber binds with bile salts, which is probably the reason for reducing blood cholesterol [79]. One more research revealed that a high-fiber-containing diet reduces insulin resistance and minimizes the risk of diabetes mellitus [81]. It has been recommended for those childhood FC cases of laxative-resistant constipation to avoid cow's milk [82]. Still, few studies have shown that a cow's milk protein (CMP)-free diet improves constipation and can be approached as a first-line treatment [83,84]. Fast-food consumption [85], increased screen time, the lack of physical activity, and obesity contribute significantly to childhood constipation [53,86-90].

The most imperative triggering factors of constipation have been identified among individuals having a low-fiber, high-calorie diet with a sedentary lifestyle that causes obesity, which is well-known as a changeable risk factor [88,90-92]. Fast food often contains high amounts of saturated and trans fat, refined sugar, salt, and phosphorus-containing preservatives [70,93-95]. These ready-to-eat meals generally comprise a meager quantity of dietary fiber [96,97]. Therefore, ready-made meal (usually processed food containing deficient fiber) promotes several health hazards [98,99] plus gastrointestinal disorders [55,75,100] such as persistent constipation [101]. It has been reported that children who spend more than two hours of screen time suffer from constipation [53,102]. Multiple studies also revealed that a scarce number of toilets in school, feeling ashamed to use standard school toilets, insufficient water consumption, and a family history of constipation also act as risks of having childhood constipation [53,90,102]. Huang et al. (2014) reported that Chinese teenagers with low-level physical endeavor had a positive association with constipation [103]. This research also revealed that primarily, those teens who are more prone to sedentary work had constipation. Additionally, this condition is reversible with increased physical effort [103]. Tantawy et al. (2017) revealed that a physical workout improves colonic motility and quickens intestinal movement passage time [104]. Multiple studies reported that regular physical drill practice improves constipation rate and minimizes its symptoms even in the elderly population [105-108].

A family history of constipation, particularly in mothers, may result in constipation in babies [88,109-111]. One study from China found that, through multiple logistic regression model analyses, nine features precipitated FC. Those are second child in birth order, children's picky eating, bad bowel habits, parental history of constipation, parents blaming the child for having a bad bowel movement, more than three hours of father-child interaction time per day, parental conflict, doting or authoritarian parenting style, and anxiety control or temper control in children [31]. However, no specific gene could be identified conclusively that may contribute to its pathophysiology [112]. Recently, several studies have reported that the rearing style of parents, especially children whose parents showed a high-autonomy attitude, developed less stool frequency [111,113]. Parents' discouraging attitude toward the development of autonomy in children decreases human internal motivation [114]. This lack of motivation causes an excessive psychological burden, raising the chance of FC [31,87]. In the present world, mental stress is a global phenomenon that results in constipation [115]. In the case of children, any kind of stress, whether home- or school-related, may act as an aggravating factor [116]. Children exposed to violence [54,117], parenteral divorce [88], or any other stressful life events [34,118] tend to develop this disorder. In the present era, in many families, both parents remain outside the home, which makes them compelled to keep their children under the supervision of caregivers [43]. As a result, toilet training may become delayed [43], and this hampers regular bowel habits [119].

Breastfeeding plays a considerable role in reducing constipation [120]. Motoki et al. (2023) conducted coast-to-coast research in Japan and revealed that an extended period (seven months or more) was contrariwise associated with the growth of FC among kids [121]. Another Japanese study by Yamakawa et al. (2015) reported that prolonged breastfeeding protects against respiratory tract infections requiring hospitalization [122]. Yamakawa et al. (2013) conducted national research regarding breastfeeding and obesity and revealed that breastfeeding is concomitant with a diminished possibility of developing overweight and obesity among Japanese school-going children [123]. One more study reported that extended breastfeeding periods minimize obesity and constipation episodes at six months, one year, and two years of age [120].

Additionally, it was also observed that with exclusive breastfeeding for up to six months, children gain a protective mechanism against acute illness [120,124]. Nakamura et al. (2021) reported that children born through lower-segment cesarean section (LSCS) were more prone to develop constipation than those born with normal vaginal delivery (NVD) [125]. Children born through NVD have been observed to create large intestinal microbiota in quality and quantity that prevent childhood FC [125-127]. There has been significantly better quality microbiome observed among children with FC than their healthy counterparts [128]. A study in Brazil found an association between FC and the usage of cow's milk in nursing bottles [46]. In their review article, Salvatore et al. commented that constipation is infrequent in breastfed children, but laxatives may be needed in nonexclusively breastfed infants [129]. Low birth weight (LBW), brief duration of breastfeeding, shorter gestation, and the timing of toilet training were associated with decreased defecation frequency by several researchers [109,119]. However, another study differs from these findings [130]. Some recent systematic reviews and meta-analysis findings are depicted in Table 1.

**Table 1:** Some important systemic reviews and meta-analysis regarding childhood and functional constipation Note: Published in the last five years (accessed on 8 December 2023) and found in the PubMed database FGIDs, functional gastrointestinal diseases; PEG, polyethylene glycol; RCTs, randomized controlled trials

Author's Name	Journal Details	Background	Result	Conclusion
Liyanarachchi et al. [116]	Neurogastroenterol Motil. 2022;34(4):e14231	Several psychological elements lead to the development of functional constipation (FC) in the pediatric age group. Among these, stressful life events also impact the development of FC	Home-related and educational institution-related stressors and mental trauma due to war and civil unrest were found to be contributors to the development of reduced stool frequency	Minor stressors at home and school and significant issues such as exposure to war are associated with FC
Hofman et al. [131]	Nutrients. 2022;14(5):974	FGIDs are common in children. Maximum FGIDs recover spontaneously with time, except for functional constipation	Altered gut microbiota is thought to play a significant part in these disorders	Except for infantile colic, data on microbiota are narrow and scarce in other FGIDs, including constipation
Wegh et al. [132]	J Pediatr. 2022;240:136-149	Parents of constipated children are interested in applying alternative medicine instead of common medications such as laxatives and PEG. Data on the impact of fluid, pro- and prebiotics, and exercise are scarce	Most of the studies showed an increased risk of biasness. Cassia fistula emulsion, avoidance of cow's milk, and abdominal electrical stimulation may be beneficial	As data were less, high-quality studies are needed to include non-pharmacological intervention in the treatment protocol
Liu et al. [133]	Front Public Health. 2021;9:663581	Functional constipation is frequently found in infants due to more extended periods and less satisfactory results. Alternative therapy is gaining attention. Traditional Chinese therapy (TCM) Infantile massage is one of them	TCM infantile massage showed significant superiority in relieving symptoms and stool frequency compared to drug only	High-quality studies are needed to reach a definite conclusion
Kamphorst et al. [134]	BMJ Paediatr Open. 2021;5(1):e001028	An altered microbiota of the intestine may contribute to the development of FGID due to immunological changes. Antibiotics may cause the disturbance of microbiota. Data are limited in the case of children	Inflammatory bowel disease (IBD) and coeliac diseases were associated with exposure to antibiotics in early childhood, but an association was not found with constipation	Treatment with antibiotics in early life may increase the risk of IBD and coeliac disease
Paknejad et al. [135]	Daru. 2019;27(2):811-826	Despite starting treatment, the minimal number of children remains symptom-free. As a result, the use of complementary and alternate medicine (CAM) is gaining attention	The majority of studies showed satisfactory results significantly	Data on the role of CAM in childhood constipation is limited. Available data showed promising results of CAM in the treatment of FC
Southwell [136]	Expert Rev Gastroenterol Hepatol. 2020;14(3):163-174	Almost one-third of children suffering from functional constipation may continue to have this problem in adulthood. New treatment options and diagnostic criteria were being sought for effective outcome	PEG is still superior to other pharmacological interventions. The diet should contain fiber, but supplementation does not help. Larger RCTs are needed to comment on probiotics	The presence of fiber in diet, the use of PEG, and the exclusion of fructose and lactulose in case of intolerance improve constipation. The use of probiotics needs high-quality RCTs

Impact of Constipation

The high prevalence of constipation and its complications has made it an urgent issue for child health [137]. It impacts children, family members, caregivers, and medical professionals [11,31,138,139]. Reduced stool frequency causes fecal incontinence (FI) [140], anal fissures [141], recurrent urinary tract infections (RUTI) [142], and enuresis in children [143]. In individuals with severe constipation, stool became solid. A stone-hard stool is rigid and bulky. Thereby, this rock-hard and inflexible stool is immovable in the rectum. This clinical condition is identified as fecal impaction. Therefore, firm and oversized stool outstretches the muscles of the large gut, especially the rectum, ultimately wearying the gut and rectum muscles. This permits watery stool further up the gastrointestinal area to pass around the impacted stool and leak out. Runny stools can seep around the hard stool and out of the bottom, causing bowel/fecal incontinence. Additionally, prolonged constipation has a chance to trigger nerve damage [144-147]. Anal fissures are mostly driven by impairment to the epithelial layer of the preceding part of the large gut (anus). Mostly, this clinical condition happens in those patients having severe cases of constipation, especially those cases having a rock-hard and oversize stool that particularly scrapes the epithelial layer of the anus [148-150]. Multiple research projects reported that pediatric patients with chronic constipation often concurrently suffer from RUTI and pyelonephritis. These patients frequently had diverse kidney and urinary tract anatomical abnormalities revealed through X-ray and ultrasonographic examination [142,151,152]. Long-standing constipation has the potential to alter the detrusor muscle activity of the urinary bladder and augment excitatory serotoninergic function, increasing the likelihood of enuresis or urine incontinence [143,153-156]. FC is considered one of the most common causes of stomachaches in the pediatric age group [157]. A study among Canadian children showed that constipation was the most frequent cause of stomachaches among kids visiting the emergency room (ER) for treatment [158]. According to a study by Zhou et al., ER visits for constipation have increased significantly in the USA [159]. Although constipation can be treated in outpatient departments (OPD), a significant portion of affected patients visit the ER, resulting in the overuse of radio imaging tests [160,161].

Several studies have shown that constipation decreases the health-related quality of life of children, parents, and caregivers [162,163]. Infrequent defecation, followed by fecal incontinence (FI), makes children stressed and causes significant psychological and emotional disturbances, which increase the risk of constipation, making it a vicious cycle [164]. This psychological stress severely impacts their psychosocial and school performance as affected children often miss school [165-167]. In one study, Rajindrajith et al. observed that affected patients suffer more from withdrawal, depression/anxiety, attention deficit, somatic complaints, and social problems [168]. Familial dysharmony and parental conflicts have also been reported to cause constipation and FI [164,168]. Early diagnosis and effective treatment can reduce the negative impact of this chronic condition [169,170]. Some recent randomized controlled trial findings are depicted in Table 2.

**Table 2:** Some important randomized controlled trials regarding childhood constipation and functional constipation (FC) Note: Published in the last five years (accessed on 8 December 2023) and found in the PubMed database

Author's Name	Journal Details	Background	Result	Conclusion
Dheivamani et al. [36]	Indian J Gastroenterol. 2021;40(2):227-233	The treatment of constipation largely includes lifestyle modification along with laxatives such as polyethylene glycol (PEG) and lactulose. PEG is suggested as the first-line drug for FC in children by clinicians	The PEG group showed higher stool frequency persistently along with reduced painful defecation in comparison with the lactulose group	PEG showed significant superiority in the treatment of FC over lactulose
van Summeren et al. [171]	Fam Pract. 2022;39(4):662-668	Low quality of life and high treatment costs because of the continuance nature of the disease make constipation a considerable burden for children and their families. Physiotherapy may help in alleviating symptoms along with expenditure if added to conventional treatment (CT)	As a first-line treatment, physiotherapy along with CT was not cost-effective, but it may be considered for chronic constipation	More eminent studies are obligatory to appraise the cost-effectiveness of physiotherapy
Qiao et al. [172]	Clin Transl Gastroenterol. 2021;12(5):e00345	Due to less efficacy and long duration of treatment, adherence to PEG is lower among patients. Parents have grown interested in Chinese herbal medicine (CHM). The safety and efficacy of XiaojiDaozhi Decoction, a component of CHM, was not well documented in FC	Patient satisfaction and complete bowel movements were significantly more significant in the CHM group than in the placebo group. The recurrence percentage was also lower in the test group	CHM XiaojiDaozhi Decoction showed superiority over the placebo group and is safe and effective
Hakimzadeh et al. [173]	Rev Gastroenterol Peru. 2019;39(4):323-328	When the hard stool is found during the digital rectal examination or fecal impaction is noticed in abdominal radiology incidentally without any obvious symptoms of constipation, the condition is known as occult constipation (OC) and is a common cause of abdominal pain (AP). Data on the efficacy of lactulose and PEG on the minimization of AP in OC are limited	Lactulose was less effective than PEG on pain reduction, although it is highly affected by age, body weight, and AP characteristics	PEG reduces AP more than lactulose, especially in older and severe cases
Arman-Asl et al. [174]	Adv Exp Med Biol. 2021;1328:411-419	The efficacy of pharmacological intervention is limited. The application of olive oil on the abdomen topically was recommended by Persian scholars, but a clinical trial was not done	Stool frequency was improved in the olive oil group in comparison to the placebo group	Olive oil is safe and effective, has no significant side effects, and can be used in childhood constipation
Yu et al. [175]	Am J Gastroenterol. 2023;118(3):553-560	Children who endure refractory constipation are thought to suffer from pelvic floor dysfunction (PFD). Pelvic floor exercises (PFE) with percutaneous tibial nerve stimulation (PTNS) have resulted in satisfactory results in aged persons, but data are limited in children	Bowel moved satisfactorily in children who received PTNS with PFE with remission of PFD. No significant unwanted effects were observed	PTNS with PFE can be considered a robust and competent treatment option in children with PFD

FC in children is responsible for significant medical spending [156,171,176,177] as they use healthcare services more than non-constipated ones and often visit emergency departments [178]. There has been no observation regarding gender as an independent risk factor for developing FC in childhood [54]. Moreover, if diagnosis is not done appropriately, it may result in frequent visits to the physician's chamber and the use of unnecessary abdominal radiographs along with other laboratory tests [179]. Research by Stephens et al. (2017) revealed that the expenditure for OPD consultation for constipation was US$120 per visit. When the patient went to the inpatient department, the cost increased to US$7815 each time of hospitalization. Many children were admitted to the inpatient department without visiting the OPD [180]. If constipation presents with complications, it may even need surgical interventions, increasing patients' treatment costs and morbidity [28,181,182].

## Conclusions

Globally, the problem of constipation is clearly on the surge. Children from developed and underdeveloped countries suffer from this functional gastrointestinal disorder. Unhealthy dietary patterns, the lack of toilet training, psychological stress, family history, and poor rearing style of children commonly predispose constipation in childhood. This chronic disorder may impact physical and psychological well-being, and the economic burden on families of children undergoing treatment is substantial. Healthcare providers need to be made aware of the proper management of these children. They should know how to impart appropriate counseling to their parents and should spread knowledge on preventive measures for the condition. It is essential to impart education to the caregivers of children regarding the importance of a balanced diet, which includes parents and other members of the community. Caregivers and parents must also be guided to encourage children to consume fruits, vegetables, and grains. Children should also be discouraged from leading a sedentary life, and physical exercise in games, which would gain the children's interest, should be promoted. Policymakers and government machinery need to address this public health concern by trying to provide a stress-free, healthy life for children. Physicians need to be encouraged to evaluate FC through the Rome IV symptom-based criteria and to promote non-pharmacological interventions for children suffering from FC since parents often fail to continue the treatment regimen. Family members should also be informed of stress's effects on children and impart a positive attitude toward them. More studies need to be carried out to understand the impact of emotional and psychological stress on children's bowel habits.
